# Previous blood pressure measurement and associated factors in student adolescents

**DOI:** 10.1590/S1679-45082015AO3358

**Published:** 2015

**Authors:** Marina Gabriella Pereira de Andrada Magalhães, Breno Quintella Farah, Mauro Virgilio Gomes de Barros, Raphael Mendes Ritti-Dias

**Affiliations:** 1 Hebiatry Graduate Program – Determiners of Health in Adolescence, Universidade de Pernambuco, Recife, PE, Brazil.

**Keywords:** Arterial pressure, Adolescent, Primary prevention, Health evaluation

## Abstract

**Objective:**

To identify prevalence of previous blood pressure measurement and analyze some associated factors in adolescents.

**Methods:**

This cross-sectional study included 6,077 adolescents aged 14 to 19 years. Demographic characteristics included (sex, age, period of study, region of residence, work, skin color, and economic) status, history of blood pressure measurement within last 12 months, local of blood pressure measurement, and reading obtained. To assess associations between previous blood pressure measurement with demographic characteristics and high blood pressure we used descriptive statistics and logistic regression analysis.

**Results:**

Out of the adolescents, 56.8% reported no blood pressure measurement within the last 12 months. The health centers and the physician’s office were most mentioned places for blood pressure measurement (28.3% and 36.9%, respectively). Boys (odds ratio of 1.64 95%CI: 1.46-1.84) aged 14 to 16 years (odds ratio of 1.12; 95%CI: 1.01-1.25), whose economic status was unfavorable (odds ratio of 1.48; 95%CI: 1.32-1.67) were significantly associated with no blood pressure measurement. Working was a protective factor for was not blood pressure measurement (odds ratio of 0.84; 95%CI: 0.73-0.97).

**Conclusion:**

Most of adolescents did not have their blood pressure measured within the last 12 months. Boys aged 14 to 16 years and those with unfavorable economic status had higher chance of not having their blood pressure measured.

## INTRODUCTION

Worldwide, high blood pressure is estimated to cause 7.5 million deaths, which accounts for 12.8% of total of all deaths.^1^ This affection is the major risk factor for coronary artery and cerebrovascular disease.^2^ Hypertension ranged from 15.1 to 24.9%,^3^ and affects more than 60% of individuals aged 60 years or older.^4^

Although frequently diagnosed in adulthood, hypertension can begin in childhood and adolescence.^5^ Reviews studies on high blood pressure illustrate that its prevalence ranges widely and reaches up to 30% of children and adolescents.^6^

The VI Brazilian Guidelines on Hypertension recommends the early diagnosis,^7^ through adoption of blood pressure measurement as an important component of routine pediatric examination. In this sense, we consider mandatory begin annual blood pressure measurement at 3 years of age, or even before, when the child present early neonatal morbid. The measurement should follow technique and classification parameters adequate to this population age range.^8^ Other recommendation is routine measurement of blood pressure within school environment.^7,8^ However, whether this latter has been put into practice is still unclear.

A previous Brazilian study^9^ illustrated a low frequency of blood pressure measurement (28.6%) among children and adolescents aged 7 to 17 years old. However, because the study was conducted only in the capital of the State, its result cannot be extrapolated for other regions, especially for rural area where differences are seen in access to health services. Other factors associated with blood pressure measurement in adolescents remain unknown, although their identification may help to implement preventive programs for this age range.

## OBJECTIVE

To determine prevalence of previous blood pressure measurement in the last year and analyze some associated factors with this measurement in adolescents.

## METHODS

### Study design

This cross-sectional epidemiological study was part of the project entitled “The practice of physical activities and health-related risk behaviors of high-school students living in the State of Pernambuco: a temporal trend study (2006-2011)”.

### Ethical considerations

The study was approved by the Ethics Committee on Research with Human Subjects of the *Universidade de Pernambuco* CAAE: 0158.0.097.000-10. The adolescents aged between 18 and 19 years signed the Consent Form, but those under 18 years the signature of Consent Form was requested from a parent or guardian.

### Target population and sample

We included adolescents aged between 14 and 19 years of both sexes who were attending public high school in the State of *Pernambuco*. The State of *Pernambuco* can be geographically divided into five regions: metropolitan region, *Zona da Mata* (forest zone), the *agreste* (arid zone), the *sertão* (backland) and *São Francisco* region. These five regions have 658 public high schools administered by the State Government. We used the following criteria to calculate the sample size: estimated population 338,698 (according to data of Pernambuco State Department of Education), confidence interval 95%, maximum error tolerance of 2, effect of sample type of 2, and because it covered the multiple analyses of behavioral the estimated prevalence was defined in 50%. In addition, we included a percentage of 20 for possible losses and/or inappropriate complete of the questionnaire that represented a minimal sample of 5,683 individuals.

We used a two-stage conglomerate sampling to guarantee a representative sample of the target population. In the first stage the sample unit was the school, stratified by its size (small, including less than 200 students; medium, including 200 to 499 students; and large, including 500 or more students), and then by geographic region. In the second stage, groups were drawn based on shift distribution (morning/evening) and grade. Schools were drawn using random numbers generated in the Statistical Package for Social Sciences (SPSS), version 20.0 (IBM Corp, NY, United States).

### Data collection

Data was collected between May and December 2011 using the translated and adapted version of the do Global School-Based Student Health Survey proposed by World Health Organization in order to evaluate lifestyle and health risk behaviors in adolescents available at www.who.int/chp/gshs/en. This questionnaire has been widely used by epidemiological studies.^10-12^ The questionnaire was applied to in the classroom. Members of the study were trained to read questions out loud to participants in order to standardize collection procedures. A copy of the manual was made available to each team to clarify possible doubts.

### Study variables

Each participant blood pressure measurement (dependent variable) was obtained using the question: “Have you had your blood pressure checked at least once within the last 12 months?”; responses were dichotomous.

Associated factors analyzed were local of measurement of blood pressure, high blood pressure and demographic characteristics such as: sex, age, region of residence (rural or urban), work and issues related with economic status.

Local of the measurement of blood pressure was obtained using the question: “Where did you have your blood pressure checked?”. This question had the following response options: (1) physician’s office; (2) health unit; (3) school; (4) pharmacy; (5) gym; (6) other place or occasion”.

Adolescents were considered hypertensive if their systolic and/or diastolic blood pressure readings were at or above the 95th percentile for the age, sex and height. Blood pressure was measured using digital monitors with automatic deflation (Omron HEM-742), validated by adolescents,^13^ and previously calibrated. Before blood pressure measurement we requested participants to avoid vigorous exercise, tobacco use, drink alcohol and caffeinated beverages. During measurement the student should remain in silence, have an empty bladder, be comfortably seated with back supported, and legs uncrossed legs. Cuff size was approved according to arm circumferences of the adolescent; it was placed in right arm, 2 to 3cm above the cubital fossa, at the heart level and palm of the hand turned upward. We performed three readings at intervals of at least 1 minute. The average of last two readings was used to represent the student’s blood pressure.

For economical classification, we elaborated a score based on interviewees’ responses. Scores were attributed to housing condition, mother’s formal education level and real and personal property. We considered values 1 or 2; 1 for variables with low impact in economic and health status such as to have a TV, a computer, access to the internet, and 2 points for variables considered basic such as to have a toilet at home, a refrigerator, piped water and mother with more than 8 years of formal education. Based on this score the sample was stratified by median^14^ favorable and unfavorable economic status and high blood pressure.

Tabulation of data was performed using the Epi Data 3.1. To detect errors, data were double tabulated and compared through the tool “validate double entry of data” to obtain information about typing errors. All errors were corrected based on the questionnaire.

The SPSS, version 20.0, was used for analyses and also descriptive statistics procedures (frequency distribution) and association measures (binary logistic regression). The frequency of previous measures with demographic variables and high blood pressure were compared by χ^2^ test. These analyses investigated possible confusing factors and identified statistical adjustment needed for multiple analyses.

We used binary logistic regression, which was characterized by odds ratio values, to evaluate associations between previous measures of blood pressure, demographic variables (sex, age range, skin color, period of study, region of residence, work and economic status) and high blood pressure. All demographic variables with p<0.20 in crude analyses were tested in adjusted model, and remained in the same level regardless of the significance level that was established in 5%.

## RESULTS

In 2011, 11,849 adolescents were enrolled in 84 schools selected in our study. However, 2,455 of them abandoned the school, 1,866 were absent in the day of data collection, and 333 refused to participate in the study (317 students and 16 parents/guardians). In addition, we excluded participants younger than 14 years old and older than 19 years old (931 adolescents), those who did not follow the recommendation for blood pressure collection or measurement and those who left the questionnaire incomplete (n=187). The final sample included 6,077 adolescents. Their demographic characteristics are shown in table 1.

**Table 1:** General characteristics of students attending public high school in the State of Pernambuco, Brazil

	All	Boys	Girls
	n (%)	n (%)	n (%)
Age range, years			
14-16	2,930 (52.6)	1,059 (48.6)	1,871 (55.2)
17-19	2,642 (47.4)	1,121 (51.4)	1,519 (44.8)
Period of study			
Morning	4,366 (71.8)	1,669 (69.0)	2,697 (73.8)
Evening	1,711 (28.2)	750 (31.0)	958 (26.2)
Region of residence			
Urban	4,509 (74.5)	1,802 (74.6)	2,705 (74.5)
Rural	1,541 (25.5)	612 (25.4)	928 (25.5)
Work			
Yes	4,713 (77.8)	774 (32.1)	572 (15.7)
No	1,348 (22.2)	1,638 (67.9)	3,074 (84.3)
Skin color			
White	1,561 (25.8)	635 (26.3)	926 (25.4)
Non-white	4,496 (74.2)	1,777 (73.7)	2,716 (74.6)
Economic status			
Unfavorable	3,722 (61.2)	1,347 (55.7)	2,372 (64.9)
Favorable	2,355 (38.8)	1,072 (44.3)	1,283 (35.1)

Boys from urban area whose economic status was favorable reported to have their blood pressure measured at a physician’s office (p<0.05). On the other hand, older girls from rural area whose economic status was unfavorable often reported to have their blood pressure measured at a health unit (p<0.05).

Local where blood pressures were measured according to characteristics of adolescents are shown in table 2.

**Table 2:** General characteristics of students attending public high school in State of Pernambuco and description of local where previous blood pressure measurement was taken within the last 12 months

Variables	Physician’s office	Health unit	School	Pharmacy	Gym	Other
	n (%)	n (%)	n (%)	n (%)	n (%)	n (%)
Sex						
Male	475 (39.3)	298 (24.7)	42 (3.5)	56 (4.6)	22 (1.8)	315 (26.1)
Female	707 (35.8)	604 (30.6)	74 (3.7)	151 (7.6)	16 (0.8)	424 (21.5)
p value	0.045	<0.001	0.695	0.001	0.011	0.003
Age range, years						
14-16	545 (37.7)	364 (25.2)	63 (4.4)	90 (6.2)	18 (1.2)	366 (25.3)
17-19	532 (36.9)	442 (30.7)	48 (3.3)	103 (7.1)	17 (1.2)	300 (20.8)
p value	0.658	0.001	0.151	0.323	0.871	0.004
Skin color						
White	885 (37.9)	650 (27.8)	83 (3.6)	145 (6.2)	31 (1.3)	540 (23.2)
Non-white	294 (35.0)	245 (29.2)	33 (3.9)	62 (7.4)	7 (0.8)	199 (23.7)
p value	0.133	0.467	0.622	0.240	0.258	0.744
Region of residence						
Rural	245 (32.2)	304 (39.9)	27 (3.5)	36 (4.7)	2 (0.3)	148 (19.4)
Urban	930 (38.6)	593 (24.6)	88 (3.7)	171 (7.1)	36 (1.5)	590 (24.5)
p value	0.001	<0.001	0.886	0.021	0.006	0.004
Work						
Yes	280 (37.6)	222 (29.9)	16 (2.2)	58 (7.8)	7 (0.9)	161 (21.6)
No	903 (37.1)	676 (27.8)	99 (4.1)	147 (6.0)	31 (1.3)	577 (23.7)
p value	0.797	0.276	0.014	0.088	0.464	0.241
Economic status						
Favorable	531 (39.5)	257 (19.1)	53 (3.9)	83 (6.2)	25 (1.9)	396 (29.4)
Unfavorable	652 (35.4)	646 (35.1)	63 (3.4)	124 (6.7)	13 (0.7)	343 (18.6)
p value	0.019	<0.001	0.44	0.523	0.301	<0.001
High blood pressure						
Present	82 (37.6)	57 (26.1)	6 (2.7)	13 (6.0)	1 (0.5)	59 (27.1)
Absent	1101 (37.1)	846 (28.5)	110 (3.8)	194 (6.5)	37 (1.2)	680 (22.9)
p value	0.878	0.456	0.468	0.74	0.301	0.161

In crude analysis, we observed a significant association between no blood pressure measurement and sex, and between work and economic status (p<0.05). However, no significant association was observed among no blood pressure measurement, age range, skin color, region of residence, high blood pressure (p<0.20) and period of study (p>0.20).

**Figure 1:**
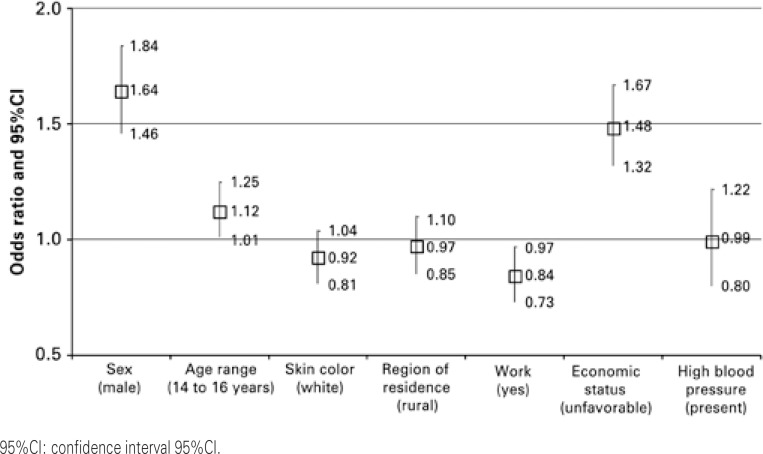
Adjusted association between demographic variables and high blood pressure with no blood pressure measurement within the last 12 months as a dependent variable

Adjusted associations among previous measure of blood pressure, demographic variables and presence of high blood pressure are presented in figure 1. We observed through an adjusted model that boys aged 14 to 16 years who did not work and whose economic status was unfavorable had more chance of do not have their blood pressure measured. No significant association was seen related with high blood pressure associated with skin color and region of residence (p>0.05).

## DISCUSSION

The main results of this study were: (i) more than half of adolescent did not have their blood measured within the last 12 months; (ii) factors associated to high chance of no blood pressure measurement were boys, aged 14 and 16 years, who did not work and had unfavorable economic status; (iii) in 34.7% of reports, blood pressure was not measured by health professionals who could identify hypertension; and (iv) local where the blood pressure was measured seemed to be related with sex, region of residence and economic status.

Positive aspects of this research were inclusion of students from both morning and evening periods and rural and urban areas; these facts were presented as limitations in previous studies. Our sample was also representative and included schools throughout the State. This approach enabled not only to be aware about the status of specific region, but also about the status of the State of Pernambuco as a whole.

Results of not measured blood pressured was higher in our study than those reported in a previous investigation conducted in Belo Horizonte (MG) Southeast Brazil,^15^ including 1,005 adolescents aged 6 to 18 years who had their blood pressure measured within the last year as recommended by health organizations.^7,8^ This finding can be explained by inclusion of younger individuals, particularly because blood pressure measurement seems to be significantly associated with age. Other study carried out in the city of Maceió (AL) Northeast Brazil,^9^ including children and adolescents observed that students who reported to have had previous blood pressure measurement were at an older age range 49%. Additionally, another study^16^ observed that frequency of blood pressure measurement increased with age and can be related with early appearance of organic disorders because of unhealthy life habits that may also increase the demand for health services among individuals.^17^ Inclusion of adolescent at an older age range in the job market may also influence the increase of demand for health services because current Brazilian Labor Law encourages periodic health evaluation for employees (annual check-ups for those younger than 18 years). Perhaps, this fact explain the reason why adolescents who work are more likely to have had their blood pressured measured.^18^

Other contributing factor for non-incorporation of the blood pressure measurement in the healthcare delivered to younger adolescents is that some procedures are need to guarantee the accuracy of the measurement, such as: choose proper cuff size for the child/adolescent’s arm, use of growth curves to identify height percentile according to age and sex, and percentile identification. These procedures require adequate devices and more time than adult blood pressure reading.^7^

Boys had less chance to have their blood pressure measured at least once than girls. A study performed by the Brazilian Institute of Geography and Statistics (IBGE - *Instituto Brasileiro de Geografia e Estatística*) including adolescents observed that women mentioned to use health services more frequently than men, regardless of their age.^19^ This finding can be explained by the number of consultations offered related with maternity or preventive actions for women in healthcare services.

A significant finding of our study was that 34.7% of blood pressure measurements were not taken at place that could identify systemic blood pressure, *i.e., *physicians’ offices and health units. This probably suggests, in most of cases, that blood pressure measurement did not result in any specific action to improve health. In addition, it also illustrates the scarcity of preventive actions at primary and secondary health services for children and adolescents. Recently, some public health programs have been implemented with actions to assist children and adolescents. For example, the school health program from the Brazilian healthcare agencies^20^ aiming to promote preventive care among scholars. This program has several preventive actions and blood pressure measurement is one of them. It might be the most effective way to offer effective health actions for children and adolescents, particularly in State of Pernambuco that 95.9% of adolescents aged 10 to 14 years are students as well as 68.5% of those aged 15 to 19 years.^19^ The mechanism of healthcare service is another area that must be enhanced considering that blood pressure measurement at adequate health service was reported only by 3.7% of adolescents.

Boys living at urban area and with favorable economic status mentioned more frequently the physician’s office as the place where they had their blood pressure measured. This finding can be explained by the concentration of physicians’ offices and healthcare professionals in less developed areas.^21^ In addition, users of Brazil Unified Health System (SUS *Sistema Único de Saúde*) compared with users of private health system has 3.8 times less physicians available, and, for this reason, those who can afford private care have more access to medical consultation,^21^ so that justifying the economic profile of boys who had more access to appropriate healthcare. The high number of boys seeking for medical consultation can be related with health evaluations required for those who work, considering that job positions in Brazil, regardless of the age, are often fulfilled by men.^22^

Older girls living in rural areas and with unfavorable economic status mentioned more frequently the health unit as the place where they had their blood pressure measured. This finding agrees with a previous study,^18^ based on data from IBGE, that illustrated that services funded by SUS, such as health units, are commonly located outside urban area in order to assist the more underserved population. In our study, as described above, a high demand for preventive health service was observed among women older than 15 years.

Higher levels of blood pressure were not associated with previous blood pressure measurement, therefore, suggesting that adolescents who need more health care attention might not have access to preventive actions such as blood pressure measurement. This is a worrying finding because cardiovascular disease commonly appears early in life.^23-26^ In addition, early identification of systemic blood hypertension enables intervention to reduce deleterious effect of hypertension.^27^

### Limitations

Limitations of this study were the lack of adolescents who were not students and students from private schools. For this reason, a selection biased should not be disregarded, because this lack can be under or overestimated our findings. The use of self-reported information regarding previous blood pressure measurement can also give space for inaccurate information. Another significant factor not included in our study was participants’ medication and medical history, however these two factors may cause confusion in the analysis.

## CONCLUSION

Results indicated that most of adolescents did not have their blood pressure measured in the last year before our study. Boys aged 14 and 16 years who were not working, and had unfavorable economic status were more likely to not have blood pressured measured at least once. Higher levels of blood pressure were not significant associated with frequency of blood pressure measurement, and, perhaps is explained for the lack of diagnosis.
