# Supplementary material for: Previous blood pressure measurement and associated factors in student adolescents
Source: Einstein (Sao Paulo). 2015 Jul-Sep;13(3):381–7. doi: 10.1590/S1679-45082015AO3358 (PMC4943783; doi:10.1590/S1679-45082015AO3358)
Supplement: Translation

# Translation: Medida prévia da pressão arterial e fatores associados em adolescentes estudantes

**Authors:** Marina Gabriella Pereira de Andrada Magalhães, Breno Quintella Farah, Mauro Virgilio Gomes de Barros, Raphael Mendes Ritti-Dias

**Affiliations:** 1 Programa de Pós-Graduação em Hebiatria – Determinantes de Saúde na Adolescência, Universidade de Pernambuco, Recife, PE, Brasil.

**Keywords:** Pressão arterial, Adolescente, Prevenção primária, Avaliação em saúde

## Abstract

**Objetivo:**

Identificar a prevalência de medida prévia da pressão arterial no último ano e analisar alguns fatores associados a essa medida em adolescentes estudantes.

**Métodos:**

Estudo transversal realizado com 6.077 adolescentes entre 14 e 19 anos. Foram coletados os dados demográficos (sexo, faixa etária, turno de estudo, região de residência, trabalho, cor da pele e situação econômica) e históricos da medida da pressão arterial nos últimos 12 meses, além do local em que essa medida foi realizada e o valor de pressão arterial. Foi utilizada análise descritiva e de regressão logística para avaliar as associações entre a medida prévia da pressão arterial e os dados demográficos e a pressão arterial elevada.

**Resultados:**

No presente estudo, 56,8% responderam não ter medido a pressão arterial nos últimos 12 meses. O posto de saúde e a consulta médica foram os lugares mais mencionados (28,3% e 36,9%, respectivamente) em que foi realizada a medida da pressão arterial. Ser do sexo masculino (*odds ratio* de 1,64; IC95%: 1,46-1,84), ter idade entre 14 e 16 anos (*odds ratio* de 1,12; IC95%:1,01-1,25) e apresentar situação econômica desfavorável (*odds ratio* de 1,48; IC95%: 1,32-1,67) foram associados positivamente a não medição da pressão arterial. Trabalhar foi fator de proteção para não medição da pressão arterial *(odds ratio* de 0,84; IC95%: 0,73-0,97).

**Conclusão:**

Elevado número de adolescentes não mediu pressão arterial nos últimos 12 meses. Rapazes, adolescentes com idade entre 14 e 16 anos e com situação econômica desfavorável apresentaram maior chance de não medir a pressão arterial.

## INTRODUÇÃO

No mundo, a pressão arterial elevada é responsável por 7,5 milhões de mortes, o que representa 12,8% do total das mortes anuais.^1^ Além disso, a pressão arterial elevada é o principal fator de risco para doença da artéria coronária e cerebrovasculares.^2^ No Brasil, a prevalência da hipertensão arterial sistêmica varia de 15,1 a 24,9%,^3^ e alcança mais de 60% daqueles com idade acima de 60 anos.^4^

Embora a maior parte dos diagnósticos de hipertensão arterial sistêmica seja firmada na idade adulta, sabe-se que essa doença pode ter início na infância e na adolescência.^5^ Estudos de revisão demonstraram que a prevalência de pressão arterial elevada apresenta grande variação, alçando até 30% em crianças e adolescentes.^6^ Por isso, o diagnóstico precoce é recomendado pelas VI Diretrizes Brasileiras de Hipertensão,^7^ por meio da incorporação da medida da pressão arterial como importante componente da rotina pediátrica. Nesse sentido, considera-se obrigatória a medida da pressão arterial a partir dos 3 anos de idade, anualmente, ou antes, quando a criança apresenta antecedentes mórbidos neonatais, utilizando-se técnicas e parâmetros de classificação adequados para essa faixa etária.^8^ Há ainda a recomendação de realização rotineira da medida da pressão arterial em ambiente escolar.^7,8^ Todavia, o quanto essas recomendações têm sido realizadas na prática ainda permanece pouco conhecido.

Em estudo realizado anteriormente,^9^ foi observada baixa frequência de medida da pressão arterial (28,6%) em crianças e adolescentes com idade entre 7 e 17 anos. No entanto, por se tratar de um estudo realizado apenas na capital, não é possível extrapolar os resultados para outras regiões, principalmente do interior, onde geralmente há diferenças em relação ao acesso aos serviços de saúde. Ademais, ainda não se conhecem os fatores associados à medição da pressão arterial nos adolescentes, embora a identificação deles possa fornecer subsídios para a implantação de programas preventivos nesse grupo etário.

## OBJETIVO

Identificar a prevalência de medida prévia da pressão arterial no último ano e analisar alguns fatores associados a essa medida em adolescentes.

## MÉTODOS

### Delineamento do estudo

Trata-se de um levantamento epidemiológico transversal de base escolar e abrangência estadual, que fez parte de um projeto intitulado “Prática de atividades físicas e comportamentos de risco à saúde em adolescentes do ensino médio no Estado de Pernambuco: estudo de tendência temporal (2006-2011)”.

### Considerações éticas

Este estudo foi aprovado pelo Comitê de Ética em Pesquisa com Seres Humanos da Universidade de Pernambuco, CAAE: 0158.0.097.000-10. Os adolescentes com idade entre 18 e 19 anos assinaram o Termo de Consentimento Livre Esclarecido para participar do estudo, enquanto para os menores de 18 anos foi fornecido o Termo de Consentimento Livre Esclarecido para os pais ou responsáveis.

### População alvo e amostra

A população alvo foi constituída por adolescentes com idades entre 14 e 19 anos, de ambos os sexos, matriculados em escolas da rede pública de Ensino Médio do Estado de Pernambuco. O Estado de Pernambuco pode ser dividido geograficamente em cinco regiões: região metropolitana, Zona da Mata, agreste, sertão e a região do São Francisco. Nas cinco regiões, existem 658 escolas públicas de Ensino Médio pertencentes à rede estadual. Para o cálculo do tamanho amostral, foram utilizados os seguintes critérios: população estimada em 338.698 (segundo dados da Secretaria de Educação do Estado de Pernambuco); intervalo de confiança de 95%; erro máximo tolerável de 2 pontos percentuais; efeito de delineamento amostral de 2; e, por abranger a análise de múltiplos comportamentos, definiu-se a prevalência estimada em 50%. Além disso, foram acrescidos 20% na amostra para possíveis perdas e/ou preenchimento inadequado do questionário, representando uma amostra mínima de 5.683 sujeitos.

Para garantir que a amostra selecionada representasse a população alvo, a seleção da amostra foi realizada por meio de conglomerados em dois estágios. No primeiro, a unidade amostral foi a escola, estratificada por porte (pequenas, com menos de 200 alunos; médias, com 200 a 499 alunos; e grandes, com 500 adolescentes ou mais) e região geográfica; no segundo, foram sorteadas turmas, considerando a distribuição por turno (diurno/noturno) e série. O sorteio das escolas foi realizado por meio de números aleatórios gerados no *Statistical Package for Social Sciences* (SPSS), versão 20.0 (IBM Corp, NY, Estados Unidos).

### Coleta de dados

Para a coleta de dados, foi utilizada uma versão traduzida e adaptada do *Global School-Based Student Health Survey* proposto pela Organização Mundial da Saúde com o intuito de avaliar o estilo de vida e os comportamentos de risco à saúde em adolescentes, que está disponível www.who.int/chp/gshs/en. Tal questionário já é amplamente utilizado por estudos epidemiológicos.^10-12^ Os dados foram coletados entre maio e dezembro de 2011. A aplicação do questionário foi realizada na sala de aula dentro da escola. Todas as questões do questionário foram lidas pelos membros da equipe, os quais foram previamente treinados, visando à padronização dos procedimentos da coleta. Além disso, um manual ficou disponível para cada equipe para eventuais dúvidas.

### Variáveis do estudo

A medida da pressão arterial no último ano (variável dependente) foi obtida por meio da pergunta: “Durante os últimos 12 meses, sua pressão arterial foi aferida (medida) pelo menos uma vez?”, sendo a resposta dicotômica.

Os fatores associados analisados foram o local da medida da pressão arterial, pressão arterial elevada e as características demográficas, como: sexo, idade, região de residência (rural ou urbana), trabalho e questões referentes à condição econômica.

O local da medida da pressão arterial foi obtido pela pergunta: “Em que local a pressão arterial foi aferida (medida)?”. Nessa questão, foram dadas como opção de resposta: “(1) consulta médica; (2) posto de saúde; (3) escola; (4) farmácia; (5) academia de ginástica; (6) em algum outro local ou ocasião”.

A pressão arterial elevada foi definida como pressão arterial sistólica e/ou diastólica maior ou igual ao percentil 95 para idade, sexo e estatura. A medida da pressão arterial foi realizada por meio de monitores digitais com deflação automática (Omron HEM-742), validados para adolescentes,^13^ previamente calibrados. Para a realização da medida, foi requerido que os adolescentes evitassem a prática de exercício físico vigoroso, fumo e ingestão de bebidas alcoólicas e cafeína. Durante a coleta, era solicitado que os alunos não estivessem com a bexiga cheia, sentassem com o dorso encostado na cadeira, em silêncio e com as pernas descruzadas. O tamanho do manguito foi apropriado de acordo com a circunferência do braço do adolescente, posicionado no braço direito, 2 a 3cm acima da fossa cubital, na altura do coração, com a palma da mão voltada para cima. Foram efetuadas três medidas, com o intervalo mínimo de 1 minuto entre elas, sendo utilizada como a medida da pressão arterial a média das duas últimas medidas.

Para a classificação econômica, foi elaborado, com base nas respostas dos entrevistados, um escore pelos pesquisadores, no qual foram atribuídas pontuações de acordo com as condições de moradia, escolaridade da mãe e posse de bens. Foram atribuídos valores de 1 ou 2, sendo 1 ponto atribuído para variáveis consideradas de pouco impacto na situação econômica e de saúde, como a posse de televisão, computador e *internet*, e 2 pontos para varáveis consideradas de necessidade básica e mais importantes, como possuir, na residência, vaso sanitário, geladeira, água encanada e escolaridade da mãe maior que 8 anos de estudo. Com base nesse escore, foi possível estratificar a amostra pela mediana:^14^ situação econômica favorável e desfavorável, e a pressão arterial elevada.

A tabulação dos dados foi efetuada no programa Epi Data 3.1. Para detectar erros, os dados foram inseridos por tabulação dupla e foi comparado por meio da ferramenta “validar dupla digitação” do programa, gerando informações sobre os erros de digitação. Todos os erros foram corrigidos com base no questionário.

As análises dos dados foram efetuadas no programa no SPSS, versão 20.0. Foram utilizados procedimentos de estatística descritiva (distribuição de frequência) e medidas de associação (regressão logística binária). O teste χ^2^ foi utilizado para comparar a frequência da medida prévia às variáveis demográficas e a pressão arterial elevada. Essas análises foram realizadas a fim de explorar possíveis fatores de confusão e para identificar a necessidade de ajustamento estatístico das análises múltiplas.

A regressão logística binária, representada pelos valores de *odds ratio*, foi utilizada para avaliar as associações entre a medida prévia da pressão arterial e as variáveis demográficas (sexo, faixa etária, cor da pele, turno de estudo, região da residência, trabalho e situação econômica) e pressão arterial elevada. Todas as variáveis demográficas com p<0,20 nas análises brutas foram testadas no modelo ajustado, permanecendo no mesmo independentemente do nível de significância, que foi estabelecido em 5%.

## RESULTADOS

No ano de 2011, 11.849 adolescentes estavam matriculados nas 84 escolas selecionadas sorteadas do estudo. No entanto, 2.455 adolescentes abandonaram a escola e 1.866 não estavam presentes no dia da coleta de dados, sendo elegíveis 7.528 adolescentes. Destes, 333 recusaram-se a participar do estudo (317 alunos e 16 pais/responsáveis); assim, 7.195 adolescentes foram avaliados. Após a exclusão dos respondentes com idade menor que 14 anos e maior que 19 anos (931 adolescentes), dos que não seguiram as recomendações para a coleta da pressão arterial ou não mediram, e os questionários incompletos (n=187), a amostra final foi composta por 6.077 adolescentes. Na tabela 1, são apresentadas as características demográficas dos adolescentes.

**Tabela 1:** Características gerais dos adolescentes do Ensino Médio da rede pública estadual de ensino de Pernambuco, Brasil

	Todos	Rapazes	Moças
	n (%)	n (%)	n (%)
Faixa etária, anos			
14-16	2.930 (52,6)	1.059 (48,6)	1.871 (55,2)
17-19	2.642 (47,4)	1.121 (51,4)	1.519 (44,8)
Turno de estudo			
Diurno	4.366 (71,8)	1.669 (69,0)	2.697 (73,8)
Noturno	1.711 (28,2)	750 (31,0)	958 (26,2)
Local de moradia			
Urbana	4.509 (74,5)	1.802 (74,6)	2.705 (74,5)
Rural	1.541 (25,5)	612 (25,4)	928 (25,5)
Trabalho			
Sim	4.713 (77,8)	774 (32,1)	572 (15,7)
Não	1.348 (22,2)	1.638 (67,9)	3.074 (84,3)
Cor da pele			
Branca	1.561 (25,8)	635 (26,3)	926 (25,4)
Não branca	4.496 (74,2)	1.777 (73,7)	2.716 (74,6)
Situação econômica			
Desfavorável	3.722 (61,2)	1.347 (55,7)	2.372 (64,9)
Favorável	2.355 (38,8)	1.072 (44,3)	1.283 (35,1)

A consulta médica foi o local de realização da medida da pressão arterial mais apontado por rapazes, residentes da área urbana e que possuíam situação econômica favorável (p<0,05). Já o posto de saúde foi apontado com mais frequência pelas mulheres, mais velhas, da região rural e com situação econômica desfavorável (p<0,05).

Na tabela 2 são apresentados os locais de realização da medida da pressão arterial de acordo com as características dos adolescentes.

**Tabela 2:** Características gerais e o local onde foi realizada a medida prévia da pressão arterial nos últimos 12 meses dos adolescentes do Ensino Médio do Estado de Pernambuco

Variáveis	Consulta médica	Posto de saúde	Escola	Farmácia	Academia de ginástica	Outro
	n (%)	n (%)	n (%)	n (%)	n (%)	n (%)
Sexo						
Masculino	475 (39,3)	298 (24,7)	42 (3,5)	56 (4,6)	22 (1,8)	315 (26,1)
Feminino	707 (35,8)	604 (30,6)	74 (3,7)	151 (7,6)	16 (0,8)	424 (21,5)
Valor de p	0,045	<0,001	0,695	0,001	0,011	0,003
Faixa etária, anos						
14-16	545 (37,7)	364 (25,2)	63 (4,4)	90 (6,2)	18 (1,2)	366 (25,3)
17-19	532 (36,9)	442 (30,7)	48 (3,3)	103 (7,1)	17 (1,2)	300 (20,8)
Valor de p	0,658	0,001	0,151	0,323	0,871	0,004
Cor da pele						
Não branca	885 (37,9)	650 (27,8)	83 (3,6)	145 (6,2)	31 (1,3)	540 (23,2)
Branca	294 (35,0)	245 (29,2)	33 (3,9)	62 (7,4)	7 (0,8)	199 (23,7)
Valor de p	0,133	0,467	0,622	0,240	0,258	0,744
Local de moradia						
Rural	245 (32,2)	304 (39,9)	27 (3,5)	36 (4,7)	2 (0,3)	148 (19,4)
Urbana	930 (38,6)	593 (24,6)	88 (3,7)	171 (7,1)	36 (1,5)	590 (24,5)
Valor de p	0,001	<0,001	0,886	0,021	0,006	0,004
Trabalho						
Sim	280 (37,6)	222 (29,9)	16 (2,2)	58 (7,8)	7 (0,9)	161 (21,6)
Não	903 (37,1)	676 (27,8)	99 (4,1)	147 (6,0)	31 (1,3)	577 (23,7)
Valor de p	0,797	0,276	0,014	0,088	0,464	0,241
Condição econômica						
Favorável	531 (39,5)	257 (19,1)	53 (3,9)	83 (6,2)	25 (1,9)	396 (29,4)
Desfavorável	652 (35,4)	646 (35,1)	63 (3,4)	124 (6,7)	13 (0,7)	343 (18,6)
Valor de p	0,019	<0,001	0,44	0,523	0,301	<0,001
Pressão arterial elevada						
Presença	82 (37,6)	57 (26,1)	6 (2,7)	13 (6,0)	1 (0,5)	59 (27,1)
Ausência	1101 (37,1)	846 (28,5)	110 (3,8)	194 (6,5)	37 (1,2)	680 (22,9)
Valor de p	0,878	0,456	0,468	0,74	0,301	0,161

Na análise bruta, foi observada associação significante entre a não medição da pressão arterial com o sexo, o trabalho e a situação econômica dos adolescentes (p<0,05). Por outro lado, não houve associação significante entre a não medição da pressão arterial com faixa etária, cor da pele, região de residência, pressão arterial elevada (p<0,20) e turno de estudo (p>0,20).

Na figura 1 são apresentadas as associações ajustadas entre a medida prévia da pressão arterial e as variáveis demográficas e presença de pressão arterial elevada. Por meio do modelo ajustado, verificou-se que rapazes, com idade entre 14 e 16 anos, que não trabalhavam e com situação econômica desfavorável apresentaram maior chance de não medir a pressão arterial. Por outro lado, cor da pele, região de residência e presença da pressão arterial elevada não apresentaram associação significante (p>0,05).

**Figura 1:** Associação ajustada entre variáveis demográficas e pressão arterial elevada com a não medição da pressão arterial nos últimos 12 meses como variável dependente

## DISCUSSÃO

Os principais resultados deste estudo foram: (i) mais da metade dos adolescentes não foram submetidos à medida da pressão arterial nos últimos 12 meses; (ii) os fatores associados a maior chance de não medir a pressão arterial foram: rapazes, com idade entre 14 e 16 anos, que não trabalhavam e com situação econômica desfavorável; (iii) em 34,7% dos relatos, o local da medida da pressão arterial não ocorreu por profissionais que possibilitassem a detecção da hipertensão arterial sistêmica; e (iv) os locais onde foi realizada a medida da pressão arterial pareceram estar relacionados a sexo, região de residência e situação econômica.

Os aspectos positivos desta pesquisa estão no fato de a avaliação ter sido realizada nos períodos diurno e noturno, assim como na região rural e urbana, o que se apresentou como limitações em outros estudos. Além disso, a pesquisa contou com amostra representativa e de abrangência estadual, possibilitando uma visão não só de regiões específicas, mas de todo o cenário do Estado de Pernambuco.

Os resultados referentes a não medição da pressão arterial no presente estudo foi superior aos achados observados em estudo anterior,^15^ realizado com 1.005 adolescentes de 6 a 18 anos de Belo Horizonte (MG) na Região Sudeste do Brasil, no qual foi visto que 26,3% dos adolescentes tiveram a pressão arterial aferida no último ano, como recomendado pelas entidades de saúde.^7,8^ Isso pode ser explicado pela inclusão de indivíduos mais jovens, visto que a medição da pressão arterial parece estar positivamente associada com a idade. De fato, em estudo realizado na cidade de Maceió (AL), no Nordeste brasileiro,^9^ com crianças e adolescentes, foi observado que, dos alunos que referiram ter tido a pressão arterial medida previamente, 49% possuíam faixa etária mais elevada. Estudo anterior^16^ constatou que a frequência de medida da pressão arterial aumenta à medida que aumenta a idade, o que pode estar relacionado com o surgimento precoce de distúrbios orgânicos, advindos de hábitos de vida pouco saudáveis, fazendo com que esses indivíduos aumentem a procura pelos serviços de saúde.^17^ A inserção do adolescente de maior faixa etária no mercado de trabalho também pode influenciar na procura pelo serviço de saúde, uma vez que as leis trabalhistas atuais incentivam avaliações de saúde periódicas para os trabalhadores (anuais para menores de 18 anos), o que parece explicar o motivo pelo qual os adolescentes que trabalham têm maior chance de medir a pressão arterial.^18^

Outro fato que pode ter contribuído para a não incorporação da medida da pressão arterial no atendimento de adolescentes mais jovens é que, para a realização da medida da pressão arterial, são necessários alguns procedimentos para garantir a fidedignidade da medida, como a escolha de manguitos adequados ao braço da criança/adolescente, a utilização de curvas de crescimento para identificação do percentil de altura segundo a idade e o sexo, e a identificação do percentil, o que demanda equipamentos adequados e mais tempo do que a realização da medida em adultos.^7^

Foi observado, ainda, que rapazes apresentavam menos chance de medida da pressão arterial comparados às moças. Em pesquisa realizada pelo Instituto Brasileiro de Geografia e Estatística (IBGE), incluindo adolescentes, foi observado que mulheres referem utilizar com mais frequência os serviços de saúde comparadas aos homens, independente da faixa etária.^19^ Este fato pode ser explicado pelas consultas relacionadas à maternidade ou a ações preventivas direcionadas a mulher nos serviços de saúde.

Interessantemente, no presente estudo, foi verificado que 34,7% das medidas da pressão arterial não ocorreram em locais que possibilitassem a detecção da hipertensão arterial sistêmica, como em consultas médicas e postos de saúde. Isso sugere que, em sua maioria, nesses casos, o ato de medir a pressão arterial provavelmente não resultou em nenhuma ação específica de saúde, e que crianças e adolescentes ainda são carentes de intervenções preventivas dos serviços de Atenção Primária e Secundária. Recentemente, alguns programas de saúde pública têm surgido com ações direcionadas para atender crianças e adolescentes. Recentemente, foi criado o Programa Saúde na Escola^20^ com a finalidade de realizar atenção preventiva entre os escolares e, dentre as diversas ações preventivas, a medida da pressão arterial é realizada. Tal programa poderia ser a maneira mais eficaz para as ações de saúde para crianças e adolescentes, uma vez que 95,9% dos adolescentes com idade entre 10 e 14 anos do Estado de Pernambuco estão matriculados, assim como 68,5% dos indivíduos de 15 a 19 anos.^19^ Todavia, os mecanismos de atenção à saúde precisam ser aperfeiçoados, pois, como pode ser observado, a realização da medida da pressão arterial neste ambiente foi apontada por apenas 3,7% dos adolescentes.

A consulta médica, como local de medida da pressão arterial, foi referida com maior frequência por rapazes, residentes da área urbana e com situação econômica favorável. Este achado pode, em parte, ser decorrente da concentração de consultórios e profissionais médicos nos centros mais desenvolvidos.^21^ Outro fato a ser considerado é que, comparados ao sistema de saúde privado, os usuários do Sistema Único de Saúde (SUS) têm 3,8 vezes menos médicos; com isto, os usuários de serviços privados possuem maior acesso à consulta médica,^21^ justificando o perfil econômico encontrado. Quanto à procura maior das consultas médicas por adolescentes do sexo masculino, esta pode estar relacionada às avaliações de saúde exigidas aos trabalhadores, uma vez que a maioria dos postos de trabalho, no Brasil, independente da faixa etária, estão preenchidos por indivíduos do sexo masculino.^22^

Já o posto de saúde foi apontado com mais frequência pelas mulheres, mais velhas, da região rural e com situação econômica desfavorável. Estes dados estão em concordância com o estudo prévio,^18^ que, por meio de dados do IBGE, observou que os serviços financiados pelo SUS, como os postos de saúde, estão, em sua maioria, fora da área urbana, atendendo a população mais carente. Neste estudo, conforme já citado, é possível observar uma maior procura dos serviços de saúde por mulheres, na faixa etária maior que 15 anos, para consultas de caráter preventivo.

A presença de níveis elevados de pressão arterial não foi associada com a medida prévia da pressão arterial, sugerindo que adolescentes que necessitam de maior atenção à saúde podem não ter tido acesso a condutas preventivas, como a medida da pressão arterial. Este achado é preocupante, uma vez que a maioria das doenças cardiovasculares surge justamente nas fases iniciais da vida.^23-26^ Além disso, a detecção da hipertensão arterial sistêmica precocemente permite intervenções que o efeito deletério dessa doença seja minimizado.^27^

### Limitações

O presente estudo apresentou limitações. Não foram incluídos adolescentes que estavam fora da escola e nem aqueles que estudavam em escolas privadas. Assim, não se pode descartar a possibilidade de ter ocorrido um viés de seleção, que pode ter subestimado ou superestimado os valores encontrados. A utilização de informações autorreferidas relacionadas à medida prévia pode ter possibilitado a ocorrência de viés de informação. Também não foi questionado o histórico de consultas médicas e nem o uso de medicações, o que poderia representar fator de confusão nas análises.

## CONCLUSÃO

Os resultados apresentados neste trabalho indicaram que a maioria dos adolescentes não realizou medida da pressão arterial no último ano. Além disso, rapazes, com idade entre 14 e 16 anos, que não trabalhavam e com situação econômica desfavorável apresentaram maior chance de não medir a pressão arterial. Outro achado deste estudo foi que a presença da pressão arterial elevada não representou associação significante com a frequência da medida da pressão arterial; assim, é provável que não tenham tido a possibilidade de diagnóstico.
